# PLASMA HIGH DENSITY LIPOPROTEIN CHOLESTEROL AND RISK OF FRACTURES IN OLDER ADULTS

**DOI:** 10.1093/geroni/igac059.2727

**Published:** 2022-12-20

**Authors:** Sultana Monira Hussain, Peter Ebeling, Anna Barker, Lawrence Beilin, Andrew Tonkin, John McNeil

**Affiliations:** Monash University, Melbourne, Victoria, Australia; Monash University, Melbourne, Victoria, Australia; Monash University, Melbourne, Victoria, Australia; The University of Western Australia, Perth, Western Australia, Australia; Monash University, Melbourne, Victoria, Australia; Monash University, Melbourne, Victoria, Australia

## Abstract

The ASPirin in Reducing Events in the Elderly trial participants (ASPREE, aged >70-years Australians), for whom high-density lipoprotein cholesterol (HDL-C) levels were measured were included. Fractures included were confirmed by medical imaging and included both traumatic and pathological fractures. Fractures were confirmed by an expert review panel. Cox proportional hazards regression was used to calculate hazard ratio (HR) and 95% confidence interval (CI) for associations with fractures. Of the 16262 participants who had a plasma HDL-C measurement at baseline 1,659 experienced at least one episode of fracture over a median of 3.98 years (interquartile range, 0.02, 7.0 years). In the fully adjusted model, each mmol/L increment in HDL-C was associated with a 34% (HR 1.34, 95% CI 1.20–1.50) higher risk of fractures. The results remained similar when these analyses were stratified by sex. Sensitivity analyses demonstrated that these associations persisted when the analyses were repeated including only: 1) nontraumatic fractures, 2) participants not on osteoporotic medications, 3) participants who were never-smokers and reported that they did not drink alcohol, and 4) participants who walked outside less than 30 minutes and reported no participation in moderate/vigorous physical activity. No association was observed between non-HDL-C and fractures. This prospective observational study suggests that higher levels of HDL-C are associated with higher fracture risk. This association was independent of the common risk factors of fractures.

